# Neural activity responsiveness by maturation of inhibition underlying critical period plasticity

**DOI:** 10.3389/fncir.2024.1519704

**Published:** 2025-01-22

**Authors:** Ibuki Matsumoto, Sou Nobukawa, Takashi Kanamaru, Yusuke Sakemi, Nina Sviridova, Tomoki Kurikawa, Nobuhiko Wagatsuma, Kazuyuki Aihara

**Affiliations:** ^1^Graduate School of Information and Computer Science, Chiba Institute of Technology, Chiba, Japan; ^2^Department of Computer Science, Chiba Institute of Technology, Chiba, Japan; ^3^Department of Preventive Intervention for Psychiatric Disorders, National Center of Neurology and Psychiatry, Tokyo, Japan; ^4^Research Center for Mathematical Engineering, Chiba Institute of Technology, Chiba, Japan; ^5^Department of Mechanical Science and Engineering, Kogakuin University, Tokyo, Japan; ^6^International Research Center for Neurointelligence, The University of Tokyo, Tokyo, Japan; ^7^Department of Intelligent Systems, Tokyo City University, Tokyo, Japan; ^8^Department of Complex and Intelligent Systems, Future University, Hakodate, Hokkaido, Japan; ^9^Department of Information Science, Toho University, Chiba, Japan

**Keywords:** critical period, gamma-aminobutyric acid, spontaneous activity, inter-trial phase coherence, spiking neural network, synaptic plasticity

## Abstract

**Introduction:**

Neural circuits develop during critical periods (CPs) and exhibit heightened plasticity to adapt to the surrounding environment. Accumulating evidence indicates that the maturation of inhibitory circuits, such as gamma-aminobutyric acid and parvalbumin-positive interneurons, plays a crucial role in CPs and contributes to generating gamma oscillations. A previous theory of the CP mechanism suggested that the maturation of inhibition suppresses internally driven spontaneous activity and enables synaptic plasticity to respond to external stimuli. However, the neural response to external stimuli and neuronal oscillations at the neural population level during CPs has not yet been fully clarified. In the present study, we aimed to investigate neuronal activity responsiveness with respect to the maturation of inhibition at gamma-band frequencies.

**Method:**

We calculated inter-trial phase coherence (ITPC), which quantifies event-related phase modulations across trials, using a biologically plausible spiking neural network that generates gamma oscillations through interactions between excitatory and inhibitory neurons.

**Results:**

Our results demonstrated that the neuronal response coherence to external periodic inputs exhibits an inverted U-shape with respect to the maturation of inhibition. Additionally, the peak of this profile was consistent with the moderate suppression of the gamma-band spontaneous activity.

**Discussion:**

This finding suggests that the neuronal population's highly reproducible response to increased inhibition may lead to heightened synaptic plasticity. Our computational model can help elucidate the underlying mechanisms that maximize synaptic plasticity at the neuronal population level during CPs.

## 1 Introduction

Neural circuits are shaped by experiences to adapt to the surrounding environment, especially during early postnatal life (Hensch, 2004; Takesian and Hensch, 2013; Werker and Hensch, 2015). In particular, brain plasticity is one of the observed hallmarks during specific windows of significant brain maturational processes known as “critical periods (CPs).” CP plasticity proceeds sequentially in somatosensory, auditory, insula, amygdala, and visual areas (Reh et al., 2020). With the onset of CP plasticity, rapid brain development enables cognitive abilities such as visual and auditory functions, language acquisition, and social and emotional functions (Rice and Barone Jr, 2000; Knudsen, 2004; Kolb et al., 2013; Larsen and Luna, 2018). Accumulating evidence indicates that the maturational process of inhibitory circuits triggers the initiation of CP (Hensch, 2004, 2005; Toyoizumi et al., 2013; Takesian and Hensch, 2013; Wong-Riley, 2021).

The maturation of the inhibitory circuits alters the excitatory-inhibitory (E/I) balance during CPs (Hensch and Fagiolini, 2005; Morishita et al., 2010; Li et al., 2012; Werker and Hensch, 2015; Fang et al., 2021; Hunter et al., 2024). In particular, inhibitory factors such as gamma-aminobutyric acid (GABA) and parvalbumin-positive (PV) interneurons are crucial for CPs by facilitating an optimal E/I balance (Hensch, 2004; Takesian and Hensch, 2013). GABA, the main inhibitory neurotransmitter, controls the maturation of interneurons and accelerates CP onset (Ben-Ari et al., 2012; Le Magueresse and Monyer, 2013). PV interneurons account for approximately 40% of GABAergic inhibitory neurons and form interconnected and synchronized networks (Hensch, 2004; Le Magueresse and Monyer, 2013; Larsen and Luna, 2018; Markram et al., 2004). Such inhibition regulates neural activity, which results in an appropriate E/I balance to initiate a CP (Fagiolini and Hensch, 2000; Takesian and Hensch, 2013; Hug and Mpai, 2024).

Plasticity during CPs has received considerable attention in neuroscience for more than 60 years (Wiesel and Hubel, 1963). The primary visual cortex (V1) has been thoroughly studied because it reflects the development of neuronal plasticity, specifically the ocular dominance (OD) plasticity caused by monocular deprivation (MD) (Wiesel and Hubel, 1963; Kuhlman et al., 2013; Toyoizumi et al., 2013; Quast et al., 2023). MD during CPs strengthens the spiking response of neurons to the open eye (OD plasticity), and this shift is particularly predominant during CP (Long et al., 2005; Hensch, 2005). It has been demonstrated in a computational model that sufficient inhibition causes significant OD shifts to the open eye following MD (Toyoizumi et al., 2013). The study concluded that the maturation of inhibition changed the neuronal activity response pattern from internally spontaneous to externally driven, thereby shifting the source of learning cues to external stimuli. However, these analyses were limited to the visual system and used a single pyramidal neuron model. Consequently, neuronal oscillations at the population level remain unclear. Further validation is necessary to clarify the contribution of inhibitory maturation to population-level neuronal oscillations.

In addition to influencing the timing of CPs, the E/I balance plays a crucial role in neuronal activity, particularly in generating gamma-band oscillations. These oscillations, induced by interactions between excitatory and inhibitory neurons, support cognitive functions and are especially observed during the development of visual functions in CPs (Börgers and Kopell, 2003; Benasich et al., 2008; Lefort et al., 2009; Quast et al., 2023). Furthermore, the gamma-band activity in local circuits responds to external signals, particularly auditory and visual stimuli. For example, studies investigating auditory steady-state response (ASSR) and steady-state visual evoked potential (SSVEP) have shown that the synchrony at gamma-band frequencies strongly correlates with the consistency of external stimuli and neural responses (Tsuchimoto et al., 2011; Tada et al., 2021; Bakhtiari et al., 2023).

Sufficient inhibition alters E/I balance and regulates neuronal activity, thereby inducing synaptic plasticity and generating gamma-band oscillations in response to external stimuli (Uhlhaas et al., 2009; Faini et al., 2018; Reh et al., 2020). Based on insights from the findings of CP onset, in this study, we hypothesized that neural activity at gamma-band frequencies would become more responsive and exhibit consistent responses to external stimuli due to the maturational process of the GABAergic system. To validate this hypothesis, we quantified the neuronal population response to external inputs at different inhibition levels using the Inter-Trial Phase Coherence (ITPC) analysis on our biologically plausible spiking neural network (SNN) model, which considers the lognormal distribution of excitatory postsynaptic potentials (EPSPs). ITPC analysis quantifies event-related phase modulations across trials (Tallon-Baudry et al., 1996; Cavanagh et al., 2009). Specifically, ITPC values indicate how consistently a neural network responds to identical stimuli across trials.

## 2 Materials and methods

### 2.1 Spiking neural network model

**Figure 1:**
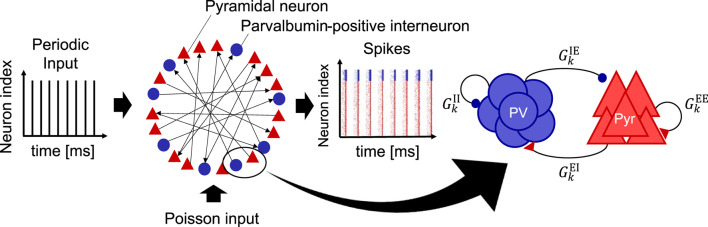
Schematic of a spiking neural network (SNN) model used in this study. This model is composed of excitatory pyramidal (Pyr) neurons (N_E = 10, 000) and parvalbumin-positive (PV) interneurons (N_I = 2, 000). This model takes into account the long-tailed distribution of excitatory postsynaptic potentials (EPSPs) for the synaptic weights from Pyr to Pyr neurons (see Figure 2). Periodic input (*f*_*s*_ = 40, 80 [Hz]) is provided to the SNN. In addition, the external input spikes according to the Poisson process with a spiking rate of 2.5 [Hz] were regularly given to all the neurons in the SNN to maintain the homeostatic firing activity. We define this sustaining activity as a spontaneous activity. To evaluate inhibitory maturation, we manipulated the synaptic weights from PV to Pyr neurons ($G_{k}^{\text{IE}}$), which is regarded as gamma-aminobutyric acid (GABA) release onto Pyr neurons. Similarly, the synaptic weights from Pyr to PV neurons ($G_{k}^{\text{EI}}$) were manipulated, regarded as glutamatergic release onto PV interneurons. In this model, the firing rate was used for evaluations, i.e., *r*_E_(*t*) (excitatory Pyr neural population) and *r*_I_(*t*) (PV neural population).

We utilized an SNN with a long-tailed distribution of EPSPs based on the model proposed by Teramae et al. (2012). All neurons were described using a leaky integrate-and-fire (LIF) model. The SNN consisted of 10, 000 excitatory pyramidal (Pyr) neurons (N_E = 10, 000) and 2, 000 inhibitory neurons (N_I = 2, 000). In our model, inhibitory neurons exclusively represent PV interneurons because PV interneurons play a pivotal role in CPs, particularly in generating gamma-band oscillations and regulating synaptic plasticity (Hensch, 2005; Werker and Hensch, 2015; Quast et al., 2023). As shown in Figure 1, periodic stimuli were input to the SNN as spike trains to evaluate stimulus-evoked neural activity. Detailed descriptions of the inputs are provided in the Supplementary material. Here, the additional spikes generated by the Poisson process were input into the SNN during all simulations to maintain the neural activity (see Figure 1).

In this study, we simulated two types of SNN activity to evaluate gamma-band neural activity: spontaneous neural activity and stimulus-evoked neural activity. The former represents spiking activity in the absence of an external periodic input and demonstrates gamma-band oscillations in our model. Spontaneous gamma-band activity typically emerges from interactions between excitatory and inhibitory neurons (Buzsáki and Wang, 2012). The latter represents spiking activity driven by periodic stimuli at gamma-band frequencies. This evoked gamma-band activity was phase-locked to the stimulus onset of each trial (Tallon-Baudry and Bertrand, 1999). For the stimulus-evoked neural activity at the gamma-band frequency, we employed periodic input frequencies *f*_*s*_ = 40 [Hz] and 80 [Hz] because these input frequencies have been utilized in physiological experiments (Tsuchimoto et al., 2011; Tada et al., 2021). Periodic input signals are provided to Pyr neurons (*j* = 1, 2, ..., N_E_) and PV interneurons (*j* = N_E_ + 1, ..., N_E_ + N_I_) in the SNN. The membrane potential of the neurons *v*_*j*_(*t*) is expressed as follows:


(1) $$\begin{array}{r} \frac{dv_{j}}{dt}=-\frac{1}{\tau_{m}}\left(v_{j}-V_{\text{L}}\right)-g_{\text{EY},j}\left(v_{j}-V_{\text{E}}\right)-g_{\text{IY},j}\left(v_{j}-V_{\text{I}}\right)\\ +\underset{i}{\sum }W_{j,i}^{\text{in}}\underset{s_{i}}{\sum }\delta \left(t-s_{i}\right), \end{array}$$

$$\text{Y}=\{\begin{array}{l} \text{E}\;\;\text{for}\;1\leq j\leq {\text{N}}_{\text{E}},\\ \text{I}\;\;\text{for}\;{\text{N}}_{\text{E}}+1\leq j\leq {\text{N}}_{\text{E}}+{\text{N}}_{\text{I}}, \end{array}$$

(2) $$\begin{array}{l} \text{if}v_{j}\left(t\right)\geq V_{\text{thr}}\text{[mV]},\;\text{then}v_{j}\left(t\right)\to V_{\text{r}}\left[\text{mV}\right], \end{array}$$

where the decay constants of the membrane τ_*m*_ are 10.5 [ms] for Pyr neurons and 3.1 [ms] for PV interneurons (Neske et al., 2015; Wagatsuma et al., 2023). The reversal potentials of the synaptic currents for the neurons and the leak current were *V*_E_ = 0 [mV] (Pyr neurons), *V*_I_ = −80 [mV] (PV interneurons), and *V*_L_ = −70 [mV]. $\underset{i}{\sum }W_{j,i}^{\text{in}}\underset{s_{i}}{\sum }\delta \left(t-s_{i}\right)$ describes the input currents generated by the input spike trains with spike time *s*_*i*_ and the input to the SNN through synaptic weights $W_{j,i}^{\text{in}}$ (see Supplementary material). Note that *s*_*i*_ spans the entire timing of the spike train from the *i*th input. Neurons in the SNN fired when the membrane potentials reached the threshold potential *V*_thr_ = −50 [mV]. Subsequently, the potentials were reset to *V*_r_ = −60 [mV] in Equation 2. According to Equation 3, Pyr neurons and PV interneurons in the SNN transmit information to each other using their conductance. The conductance of Pyr and PV neurons are represented by *g*_EY, *j*_(*t*) and *g*_IY, *j*_(*t*), respectively. Specifically, *g*_EY, *j*_(*t*) and *g*_IY, *j*_(*t*) (Y = E orI) represent the conductances of α-amino-3-hydroxy-5-methyl-4-isoxazole propionic acid (AMPA) and GABAergic synapses, respectively. The dynamics of conductance conform to the following equations (Teramae and Fukai, 2007):


(3) $$\begin{array}{l} \frac{dg_{\text{XY},j}}{dt}=-\frac{g_{\text{XY},j}}{\tau_{s}}+\underset{k}{\sum }G_{k}^{\text{XY}}\underset{s_{k}}{\sum }\delta \left(t-s_{k}-d_{k}\right), \end{array}$$

$$\text{X}=\text{E},\text{I},\;\text{Y}=\left\{ \begin{array}{l} \text{E for }1\leq j\leq {\text{N}}_{\text{E}},\\ \text{I}\;\text{for }{\text{N}}_{\text{E}}+1\leq j\leq {\text{N}}_{\text{E}}+{\text{N}}_{\text{I}}, \end{array}\right.$$

where δ(*t*) is the Dirac delta function and τ_*s*_, *s*_*k*_, and *d*_*k*_ denote the decay constants of the synaptic currents (τ_*s*_ = 2 [ms] for Pyr neurons and τ_*s*_ = 4 [ms] for PV interneurons), spiking time of the input from the *k*th neuron, and synaptic delay, respectively. $G_{k}^{\text{EE}}$, $G_{k}^{\text{EI}}$, $G_{k}^{\text{II}}$, and $G_{k}^{\text{IE}}$ represent the synaptic weights of Pyr-to-Pyr, Pyr-to-PV, PV-to-PV, and PV-to-Pyr neurons, respectively. When a spike is received at time *t* = (*s*_*k*_ + *d*_*k*_) from the *k*th presynaptic neuron, the spike weighted by $G_{k}^{\text{XY}}$ is transmitted to the *j*th postsynaptic neuron.

**Figure 2:**
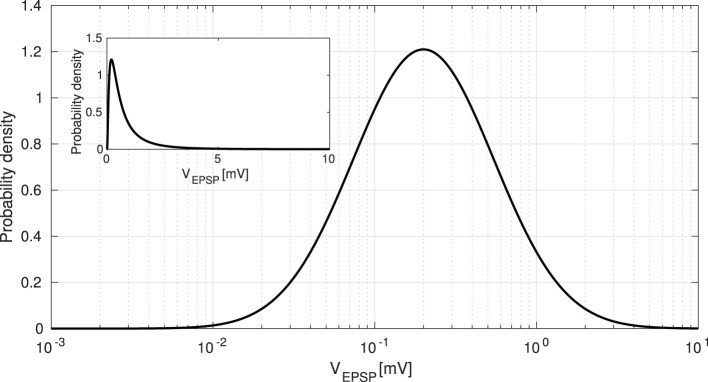
Long-tailed distribution of excitatory postsynaptic potentials (EPSPs). EPSPs are approximated as a lognormal distribution given by Equation 4. To examine the dominance of spontaneous activity, we manipulated the EPSP threshold Θ_EPSP_ from 5 [mV] to 10 [mV]. The inset shows the same distribution with the horizontal axis in a linear scale, rather than in a logarithmic scale.

As previously mentioned, our model considers the long-tailed distribution of EPSPs. Specifically, we applied this characteristic to the synaptic weights of Pyr-to-Pyr neurons ($G_{k}^{\text{EE}}$). First, EPSP amplitudes *V*_EPSP_ [mV] are generally distributed across a few large synaptic connections to many small synaptic connections (Lefort et al., 2009). This distribution can be approximated as a lognormal distribution, as shown in Figure 2. Therefore, we generate *V*_EPSP_ by using the following equation:


(4) $$\begin{array}{l} p\left(x\right)=\frac{\exp\left[-{\left(\log x-\mu \right)}^{2}/2\sigma^{2}\right]}{\sqrt{2\pi }\sigma x}, \end{array}$$

where *x* is the amplitude of the EPSPs (Teramae et al., 2012). We set σ = 1.0 and μ − σ^2^ = log(0.2) in the approximation. Second, we calculated the EPSP values to be biologically plausible (Lefort et al., 2009). If the calculated value of the EPSPs exceeded Θ_EPSP_ [mV] (*V*_EPSP_ ≥ Θ_EPSP_), the value was regenerated to be less than Θ_EPSP_ [mV] by Equation 4, following the rejected sampling method. Here, we changed the EPSP threshold Θ_EPSP_ to examine the dominance of the spontaneous activity. The lack of a few large EPSPs decreases autonomous spontaneous activity, whereas large EPSPs enhance it (Teramae et al., 2012). Specifically, we changed the EPSP threshold Θ_EPSP_ from 5 [mV] to 10 [mV] within a biologically plausible range (Lefort et al., 2009). In addition, *V*_EPSP_ is an observable value, and it must be converted into a synaptic weight for use in our model. Thus, we finally translated *V*_EPSP_ into the synaptic weight $G_{k}^{\text{EE}}$ based on a previous study, which considered the relationship between the EPSP and $G_{k}^{\text{EE}}$ as $G_{k}^{\text{EE}}=V_{\text{EPSP}}/100$ (Nobukawa et al., 2019).

### 2.2 Manipulation of synaptic weights of inhibitory-to-excitatory and excitatory-to-inhibitory neurons

As described above in Section “2.1 Spiking Neural Network Model,” we modeled the conductance of GABAergic and AMPA synapses as synaptic weights from PV neurons ($G_{k}^{\text{IY}}$) and Pyr neurons ($G_{k}^{\text{EY}}$) based on previous research (Teramae and Fukai, 2007). Specifically, in this study, we modified synaptic weights from PV-to-Pyr neurons ($G_{k}^{\text{IE}}$) and Pyr-to-PV neurons ($G_{k}^{\text{EI}}$), which are related to the degree of GABAergic neurotransmission to Pyr neurons and glutamatergic neurotransmission to PV interneurons, respectively (Teramae and Fukai, 2007).

To model GABA maturation, which plays a key role in CPs, we changed the synaptic weights from PV to Pyr neurons with $0.0017\leq G_{k}^{\text{IE}}\leq 0.0045$. In addition, we investigated the influence of reduced glutamatergic neurotransmission on PV interneurons during post-CP development. Faini et al. reported a significant reduction in glutamatergic synaptic strength in PV interneurons (Faini et al., 2018). To simulate this in our model, we decreased the Pyr-to-PV synaptic weights from the original value of $G_{k}^{\text{EI}}=0.018$ to $G_{k}^{\text{EI}}=0.013$. Furthermore, Faini et al. concluded that the strength of glutamatergic thalamic input on PV interneurons is weakened during post-CP. To account for this, we also reduced the synaptic weight $W_{j,i}^{\text{in}}$, which represents the strength between input neurons and PV interneurons, from its initial value of 0.5 [mV] to 0.1 [mV].

For the other parameters, we applied the original settings (Teramae et al., 2012). The synaptic weights were set to $G_{k}^{\text{II}}=0.0025$ (PV-to-PV), and $G_{k}^{\text{EE}}$ was followed by the distribution of EPSPs, as described above. The connection probabilities and values of the synaptic delays were set to fixed values based on previous studies (Teramae et al., 2012). The connection probabilities were set to 0.1 (Pyr-to-PV) and 0.5 (PV-to-Pyr and PV-to-PV). We randomly set the values of the synaptic delays within the ranges of 1–3 [ms] (Pyr-to-Pyr) and 0–2 [ms] (others) (Teramae et al., 2012; Nobukawa et al., 2019). We adjusted the probability of transmission failure between the Pyr neurons (Pyr-to-Pyr) as follows: *p*_E_ = *a*/(*a* + *V*_EPSP_), where *a* = 0.1 [mV] (Lefort et al., 2009; Teramae et al., 2012; Nobukawa et al., 2019). The parameters of the proposed model are listed in Table 1.

**Table 1:** Parameters for our spiking neural network.

**Parameters**	**Descriptions**	**Values**	**References for parameter values**
*V*_L_	Reversal potential of leak	–70 [mV]	Teramae et al., 2012
*V*_thr_	Threshold potential	–50 [mV]	Teramae et al., 2012
*V*_r_	Reset potential	–60 [mV]	Teramae et al., 2012
τ_m_	Decay constant of the membrane for Pyr neuron	10.5 [ms]	Wagatsuma et al., 2023
	Decay constant of the membrane for PV interneuron	3.1 [ms]	Wagatsuma et al., 2023
*V*_E_	Reversal potential of synaptic current for Pyr neuron	0 [mV]	Teramae et al., 2012
*V*_I_	Reversal potential of synaptic current for PV interneuron	–80 [mV]	Teramae et al., 2012
$W_{j,i}^{\text{in}}$	Synaptic weight after spiking from input neuron	0.5 [mV]	
τ_s_	Decay constant of the synaptic current for Pyr neuron	2 [ms]	Teramae et al., 2012
	Decay constant of the synaptic current for PV interneuron	4 [ms]	Wagatsuma et al., 2023
$G_{k}^{\text{EE}}$	Synaptic weight of Pyr-to-Pyr neurons	^*^	Teramae et al., 2012
$G_{k}^{\text{EI}}$	Synaptic weight of Pyr-to-PV neurons	^**^	Teramae et al., 2012
$G_{k}^{\text{IE}}$	Synaptic weight of PV-to-Pyr neurons	^***^	Teramae et al., 2012
$G_{k}^{\text{II}}$	Synaptic weight of PV-to-PV neurons	0.0025	Teramae et al., 2012

$*G_{k}^{\text{EE}}$ follows the long-tailed distribution. $*G_{k}^{\text{EI}}$ is changed from 0.013 to 0.018. $*G_{k}^{\text{IE}}$ is change from 0.0017 to 0.0045.

### 2.3 Evaluation methods

#### 2.3.1 Recording neuronal activity

We evaluated the neural responses of stimulus-evoked and spontaneous activities in neuronal populations in our SNN. We established a Pyr neural population consisting of 10, 000 Pyr neurons and a PV neural population consisting of 2, 000 PV interneurons in the SNN. To measure the firing activity, we determined the firing rates of each neuronal population *r*_E_(*t*) [Hz] (Pyr) and *r*_I_(*t*) [Hz] (PV) as follows:


(5) $$\begin{array}{l} r_{\text{X}}\left(t\right)=10^{3}\frac{S_{\text{X}}\left(t\right)}{\text{Δ}t},\;X=\text{E},\text{I}, \end{array}$$

where *S*_X_ denotes the number of spikes in a time bin of Δ*t* = 0.1 [ms] in each neural population. Subsequently, *r*_X_(*t*) was smoothed using a window of 1 [ms] with a Gaussian filter. In addition, we used different random seeds for connectivity between neurons during each simulation.

#### 2.3.2 Inter-trial phase coherence

ITPC was calculated for the firing rates (*r*_E_(*t*) and *r*_I_(*t*)) of the neuronal populations induced by periodic stimuli (*f*_*s*_ = 40, 80 [Hz]) to measure stimulus-induced responses. ITPC measures the event-related phase coherence for a given frequency band across trials (Cavanagh et al., 2009; Legget et al., 2017). In this analysis, ITPC values varied from 0 to 1, where 0 indicated no phase coherence across trials, and 1 indicated perfect phase coherence across trials. In other words, a higher ITPC value corresponds to a better phase coherence of neuronal activity with external signals. The ITPC across *T* trials was defined as


(6) $$\text{ITPC }(T,f)=\left|\frac{1}{T}\sum_{j=1}^{T}\frac{F_{j}(f)}{\left|F_{j}(f)\right|}\right|,$$

where *T* is the number of trials and *f* is the Fourier frequency. *F*_*j*_(*f*) is the Fourier component at frequency *f* in the *j*th trial of *r*_E_(*t*) and *r*_I_(*t*) (Li et al., 2023). In this study, the number of trials was set to *T* = 100. The reason for this parameter value is described in Supplementary material.

#### 2.3.3 Power spectrum analysis

The power spectrum of the firing rate *r*_E_(*t*) for both spontaneous neural activity and stimulus-evoked neural activity was computed to quantify neuronal oscillations. Power spectrum analysis was conducted across 10 trials. We calculated the average and standard deviation for each frequency, and compared the different synaptic weights from PV-to-Pyr neurons $G_{k}^{\text{IE}}$.

## 3 Results

### 3.1 Measuring neural activity responsiveness by maturation of inhibition

**Figure 3:**
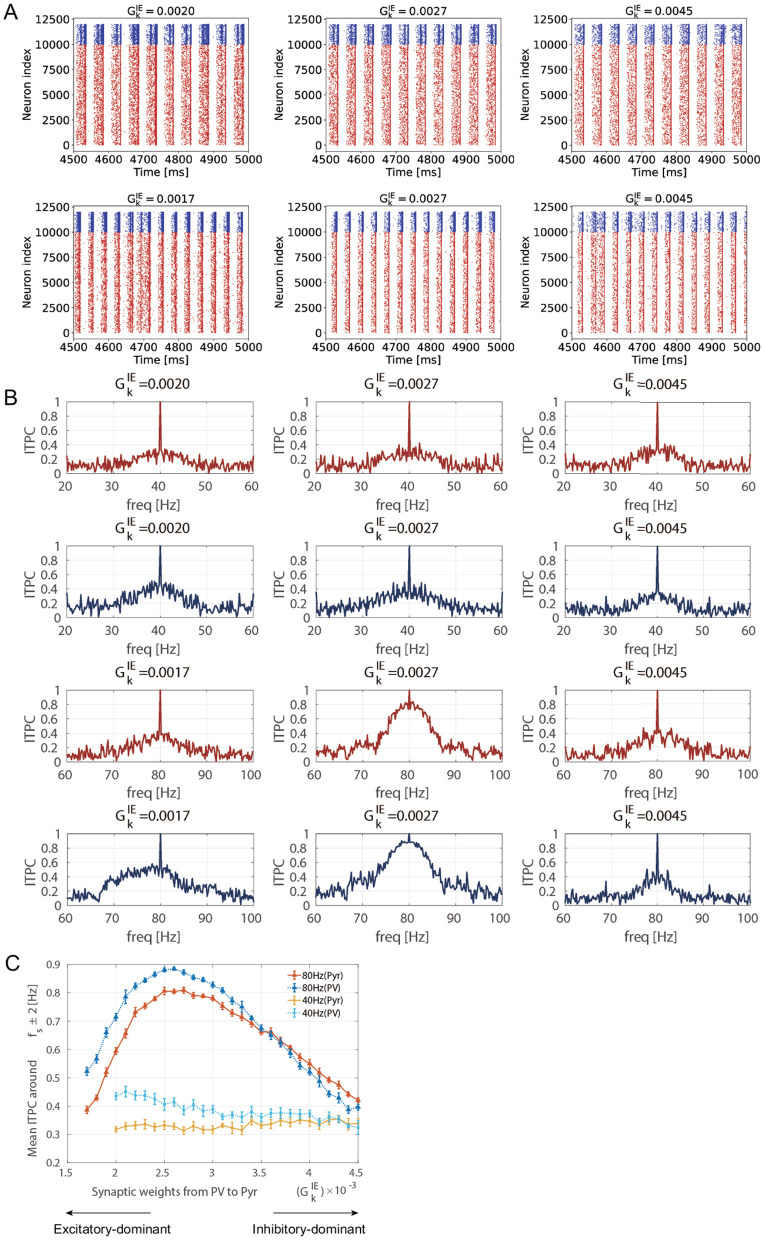
Inter-trial phase coherence (ITPC) of the firing-rate time series of excitatory pyramidal (Pyr) neuron and parvalbumin-positive (PV) interneuron populations and raster plots showing spike trains of Pyr and PV neurons in different inhibition levels $0.0017\leq G_{k}^{\text{IE}}\leq 0.0045$ (Θ_EPSP_ = 5 [mV]). **(A)** The raster plots show spike trains of Pyr (red, index from 0 to 9,999) and PV (blue, index from 10,000 to 11,999) neurons. The upper row shows the raster plots at 40 [Hz] and the lower row shows the raster plots at 80 [Hz] in different inhibition levels $G_{k}^{\text{IE}}=0.0017,0.0020,0.0027,0.0045$. **(B)** The representative ITPC profiles of different inhibition levels $G_{k}^{\text{IE}}$. The upper two rows show the ITPC profile of 40 [Hz] for Pyr (red) and PV (blue) neuronal populations. The lower two rows show the ITPC profile of 80 [Hz] for Pyr (red) and PV (blue) neuronal populations. **(C)** The mean ITPC value in the Pyr neuronal population (yellow for 40 [Hz] and orange for 80 [Hz]) and PV neuronal population (light blue for 40 [Hz] and blue for 80 [Hz]) around the input frequency *f*_*s*_ at gamma-band input frequencies {*f*_*s*_ = 40 (cross markers) and 80 (triangle markers) [Hz]} averaged *f*_*s*_±2 [Hz]. Note that we did not plot the mean ITPC at 40 [Hz] for $G_{k}^{\text{IE}}=0.0017,0.0018,$ and 0.0019 because the neural activity exhibited unrealistically high firing rates. The markers and error bars show the mean and standard deviation across 10 evaluations.

Figure 3A shows the stimulus-evoked spiking activity at 40 [Hz] and 80 [Hz] for Pyr and PV neuronal populations. The ITPC values were calculated for the firing-rate time series of these neuronal populations (*r*_E_(*t*) and *r*_I_(*t*)). Figure 3B shows the ITPC profiles at 40 [Hz] and 80 [Hz] for different levels of inhibition ($G_{k}^{\text{IE}}=0.0017,0.0020,0.0027,0.0045$). Remarkably, the ITPC value near the input frequency (*f*_*s*_±2 [Hz]) of 80 [Hz] at $G_{k}^{\text{IE}}=0.0027$ was significantly higher than at other inhibition levels ($G_{k}^{\text{IE}}=0.0017,0.0045$). In contrast, the ITPC value was not significantly influenced by the inhibition levels for an input frequency of 40 [Hz]. To quantify this characteristic of the ITPC profile, the mean ITPC values were calculated. The mean ITPC value was calculated by averaging the ITPC over the range of *f*_*s*_±2 [Hz] to quantify the neuronal coherence evoked by the stimuli. Figure 3C shows the mean ITPC values across inhibition levels for each frequency. The characteristic ITPC profile ($G_{k}^{\text{IE}}=0.0027$) at 80 [Hz] (see Figure 3B) showed significantly higher coherence (mean ITPC >0.8) than the other inhibition levels. Consequently, the mean ITPC values exhibited an inverted U-shape as inhibition increased. In contrast to the mean ITPC value at 80 [Hz], no inverted U-shape was observed at 40 [Hz]. In addition, the power of the stimulus-evoked neural activity at each input frequency (40 [Hz] and 80 [Hz]) was not influenced by inhibition levels (see Supplementary Figure S4).

**Figure 4:**
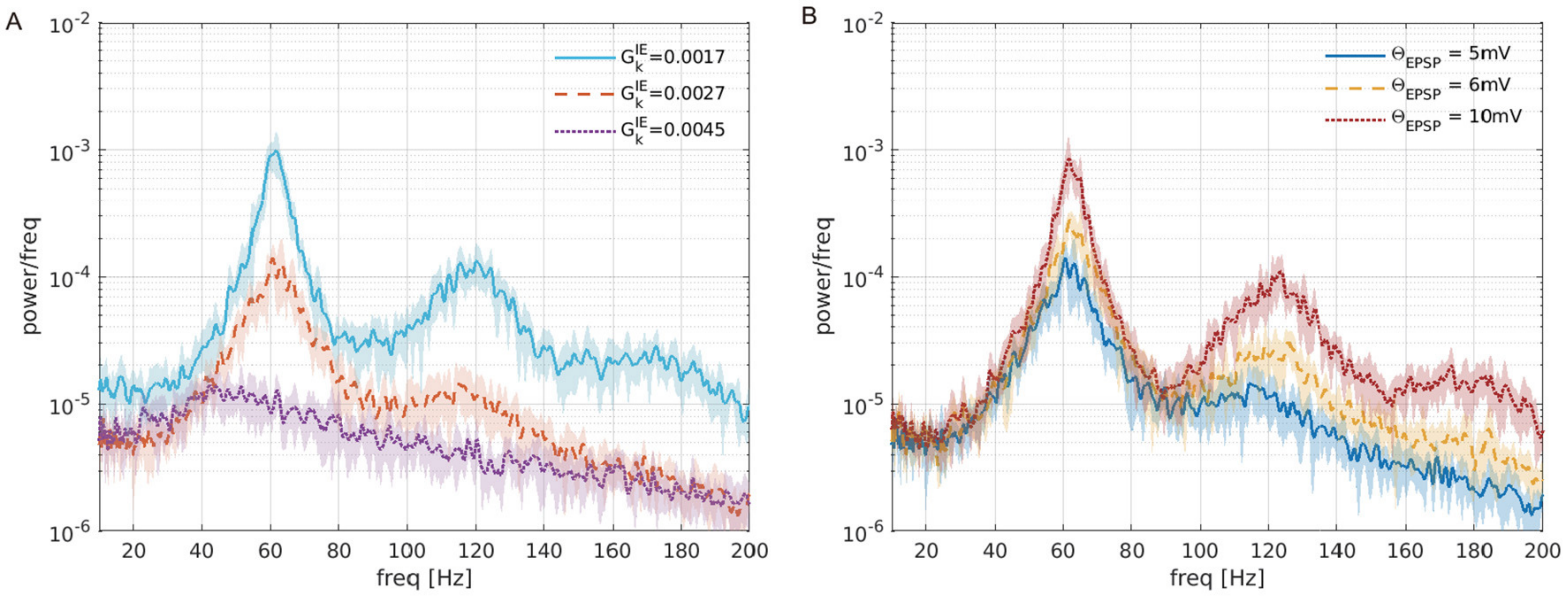
Power spectra of the firing-rate time series of the excitatory pyramidal (Pyr) neural population (*r*_E_(*t*)) in the absence of periodic input. **(A)** Different inhibition levels ($G_{k}^{\text{IE}}=0.0017,0.0027,0.0045$) with the upper threshold for EPSPs distribution set at 5 [mV] (Θ_EPSP_ = 5 [mV]). **(B)** Different EPSPs thresholds (Θ_EPSP_ = 5, 6, 10 [mV]) with the synaptic weights $G_{k}^{\text{IE}}=0.0027$. The lines and shaded areas represent the mean and standard deviation over 10 trials.

**Figure 5:**
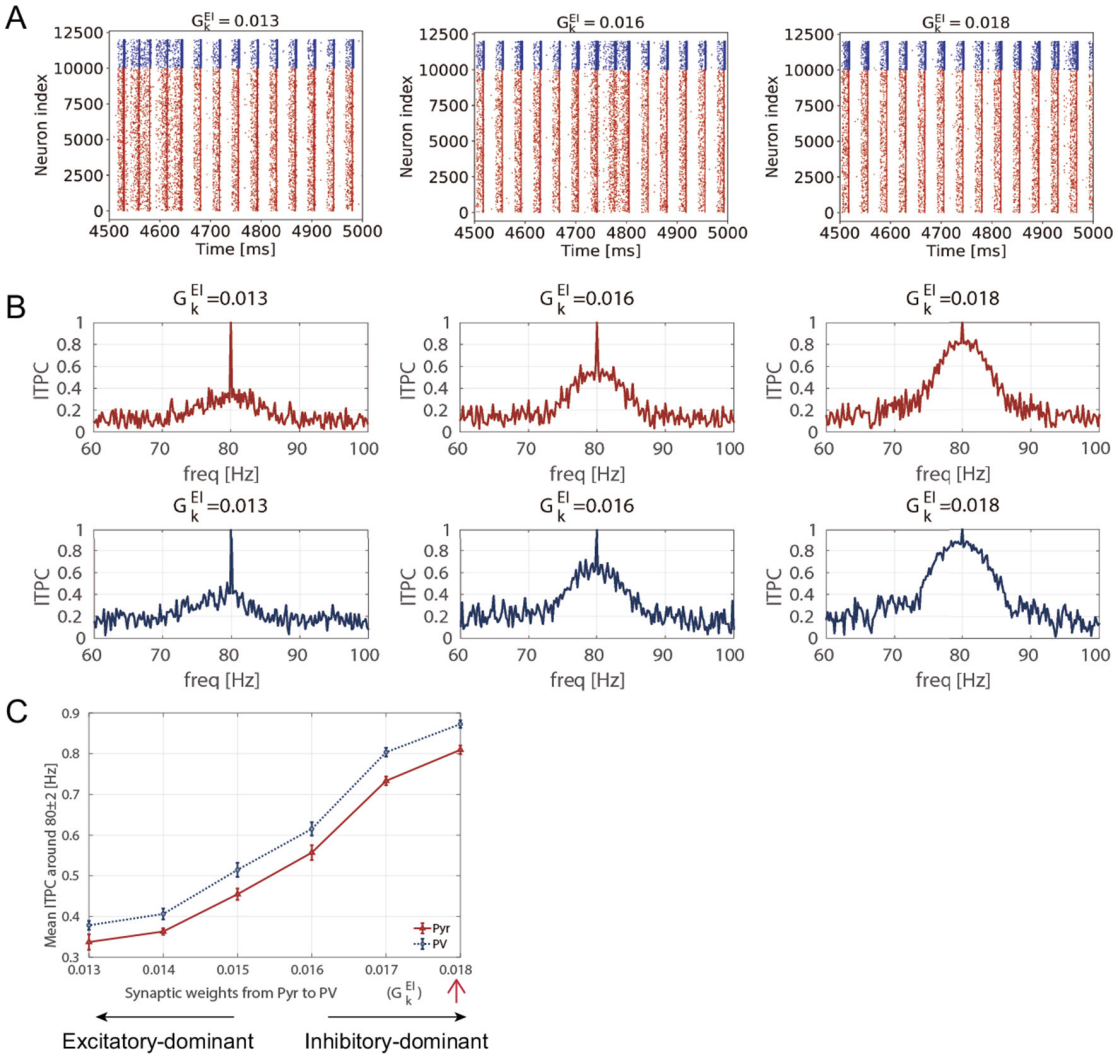
Inter-trial phase coherence (ITPC) of the firing-rate time series of excitatory pyramidal (Pyr) neuron and parvalbumin-positive (PV) interneuron populations and raster plots showing spike trains of Pyr and PV neurons within $0.013\leq G_{k}^{\text{EI}}\leq 0.018$ and $G_{k}^{\text{IE}}=0.0027$ (Θ_EPSP_ = 5 [mV]). **(A)** The raster plots show spike trains of Pyr (red, index from 0 to 9,999) and PV (blue, index from 10,000 to 11,999) neurons in different synaptic weights from Pyr-to-PV $G_{k}^{\text{EI}}=0.013,0.016,0.018$. **(B)** The representative ITPC profiles of $G_{k}^{\text{EI}}=0.013,0.016,0.018$. The two rows show the ITPC profile of 80 [Hz] for Pyr (red, upper) and PV (blue, lower) neuronal populations. **(C)** Mean ITPC values in the Pyr (red, triangle markers) and PV (blue, circle markers) neuronal populations around the input frequency *f*_*s*_ = 80 [Hz] averaged *f*_*s*_±2 [Hz]. The red arrow indicates the case where the mean ITPC value is maximized at $G_{k}^{\text{EI}}=0.018$ in Figure 3C. The markers and error bars show the mean and standard deviation across 10 evaluations.

To reveal the causes of ITPC dependency on the maturation of inhibition, we compared the spontaneous activity with the corresponding parameters of $G_{k}^{\text{IE}}$ used in the ITPC analysis ($G_{k}^{\text{IE}}=0.0017,0.0027,0.0045$). Figure 4A shows the power spectra of spontaneous activity in the case of $G_{k}^{\text{IE}}=0.0017,0.0027,0.0045$, which correspond to the excitatory-dominant (the low mean ITPC values < 0.5), balanced (the high mean ITPC >0.8), inhibitory-dominant (the low mean ITPC values < 0.5), respectively in the Figure 3C. As shown in Figure 4A, the power of the spontaneous activity decreased monotonically with the maturation of inhibition. Compared with the results at the peak of the ITPC, we found that the optimal power level of spontaneous activity contributed to the enhancement of the stimulus-evoked neuronal activity response at 80 [Hz]. Under the conditions of predominant inhibition (e.g., $G_{k}^{\text{IE}}=0.0045$), the spontaneous activity showed decreased power. Subsequently, this condition demonstrated low coherence (mean ITPC < 0.5) (see Figure 5C). These results suggest that the moderate suppression of stimulus-irrelevant spontaneous activity due to the maturation of inhibition contributes to higher coherence in the SNN at 80 [Hz]. In contrast, the same effect was not observed at 40 [Hz]. Thus, this evaluation revealed that frequency selectivity was exhibited in our SNN, depending on the inhibition levels.

As demonstrated in this evaluation, our results showed an optimized E/I balance that maximized the neuronal response coherence by manipulating the synaptic weights from PV-to-Pyr ($G_{k}^{\text{IE}}$), specifically at 80 [Hz]. Therefore, we demonstrated the results of ITPC values at 80 [Hz] in the following sections.

### 3.2 Disruption of consistency induced by E/I imbalance due to reduced Pyr-to-PV synaptic weights

To investigate the effect of the reduction in the glutamatergic synaptic strength in PV interneurons during post-CP on the neuronal response, as demonstrated by Faini et al. (2018), we analyzed the ITPC at 80 [Hz]. Specifically, we reduced the Pyr-to-PV synaptic weights from an original value of $G_{k}^{\text{EI}}=0.018$ to 0.013. Figure 5A shows the stimulus-evoked spiking activity at 80 [Hz] for Pyr and PV neuronal populations. In our SNN model, reducing $G_{k}^{\text{EI}}$ led to increased spiking activity. Figure 5B shows the ITPC profiles for representative parameter values ($G_{k}^{\text{EI}}=0.013,0.016,0.018$). As the Pyr-to-PV synaptic weights decreased, the neuronal coherence within the input frequency range (80 ± 2 Hz) was reduced monotonically, with the mean ITPC values decreasing from 0.8 to 0.3, as shown in Figure 5C. This analysis indicates that the reduction in glutamatergic synaptic strength in PV interneurons within the network decreases coherence with external inputs in our model. This finding reveals that the internal E/I ratio within neural circuits modulates neuronal responsiveness to external stimuli. A decrease in responsiveness indicates that consistent responses to input are limited, which in turn suggests that plastic changes may also be suppressed during post-CP.

**Figure 6:**
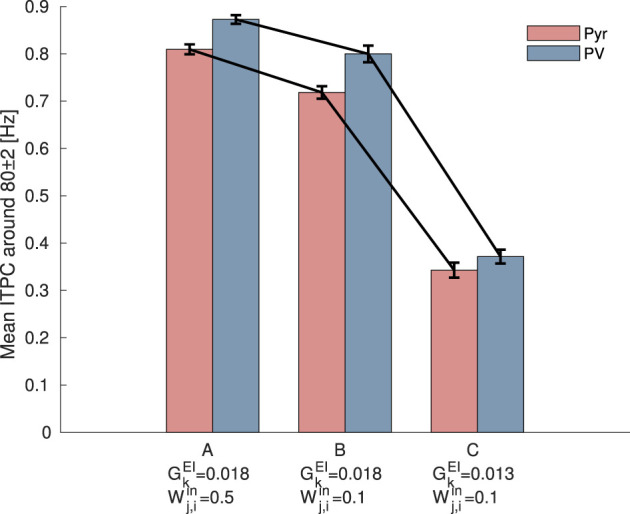
Mean inter-trial phase coherence (ITPC) values for excitatory pyramidal (Pyr) neurons (red) and parvalbumin-positive (PV) interneurons (blue) populations under different synaptic weight settings. (A) The setting corresponds to the condition before the modulation of synaptic weights, where ITPC exhibited a peak in Figure 3C. (B) The setting where the strength of external inputs to PV interneurons, $W_{j,i}^{\text{in}}$, was decreased from 0.5 [mV] to 0.1 [mV]. (C) The setting where both the synaptic weights of the external inputs to PV interneurons, $W_{j,i}^{\text{in}}$, and the synaptic weights from Pyr to PV neurons, $G_{k}^{\text{EI}}$, were decreased. Both reductions resulted in a more pronounced decrease in the mean ITPC values for 80 [Hz] input. The bars and error bars represent the mean and standard deviation across 10 evaluations.

Furthermore, to investigate the influence of the reduced strength of glutamatergic thalamic input on PV interneurons, as reported by Faini et al., we also decreased the synaptic weight $W_{j,i}^{\text{in}}$, which represents the strength between input neurons and PV interneurons, from 0.5 [mV] to 0.1 [mV]. Figure 6 shows the mean ITPC values for Pyr and PV neuronal populations under different parameter settings. The results indicate that reducing the strength of external inputs to PV interneurons causes a decrease in the mean ITPC values for both Pyr and PV populations. Furthermore, a more pronounced reduction in ITPC values is observed when $G_{k}^{\text{EI}}$ is decreased from 0.018 to 0.013, compared with the condition where it remains at 0.018. This analysis indicates that the reduction in the strength of external input on PV interneurons decreases neuronal coherence. Similar to the decrease in neuronal responsiveness caused by changes in the internal E/I ratio, it is suggested that neuronal responsiveness may also be influenced by changes in the strength of external inputs. This finding may support the study by Faini et al. (2018), which reported that the reduction of plasticity of thalamic synapses onto PV interneurons occurs during age-related development.

### 3.3 Disruption of consistent responses to input stimuli by predominant spontaneous neural activity

We further investigated the effects of strong EPSPs on ITPC values. As mentioned previously, the distribution of EPSPs was long-tailed (see Figure 2), and it induced spontaneous activity due to the coexistence of a few large and many small EPSPs (Teramae et al., 2012). In particular, the existence of a few large EPSPs boosted neuronal firing, possibly resulting in impaired cognitive function (Obi-Nagata et al., 2023). As shown in Figure 4B, such strong EPSPs had larger power spectra across all frequencies.

**Figure 7:**
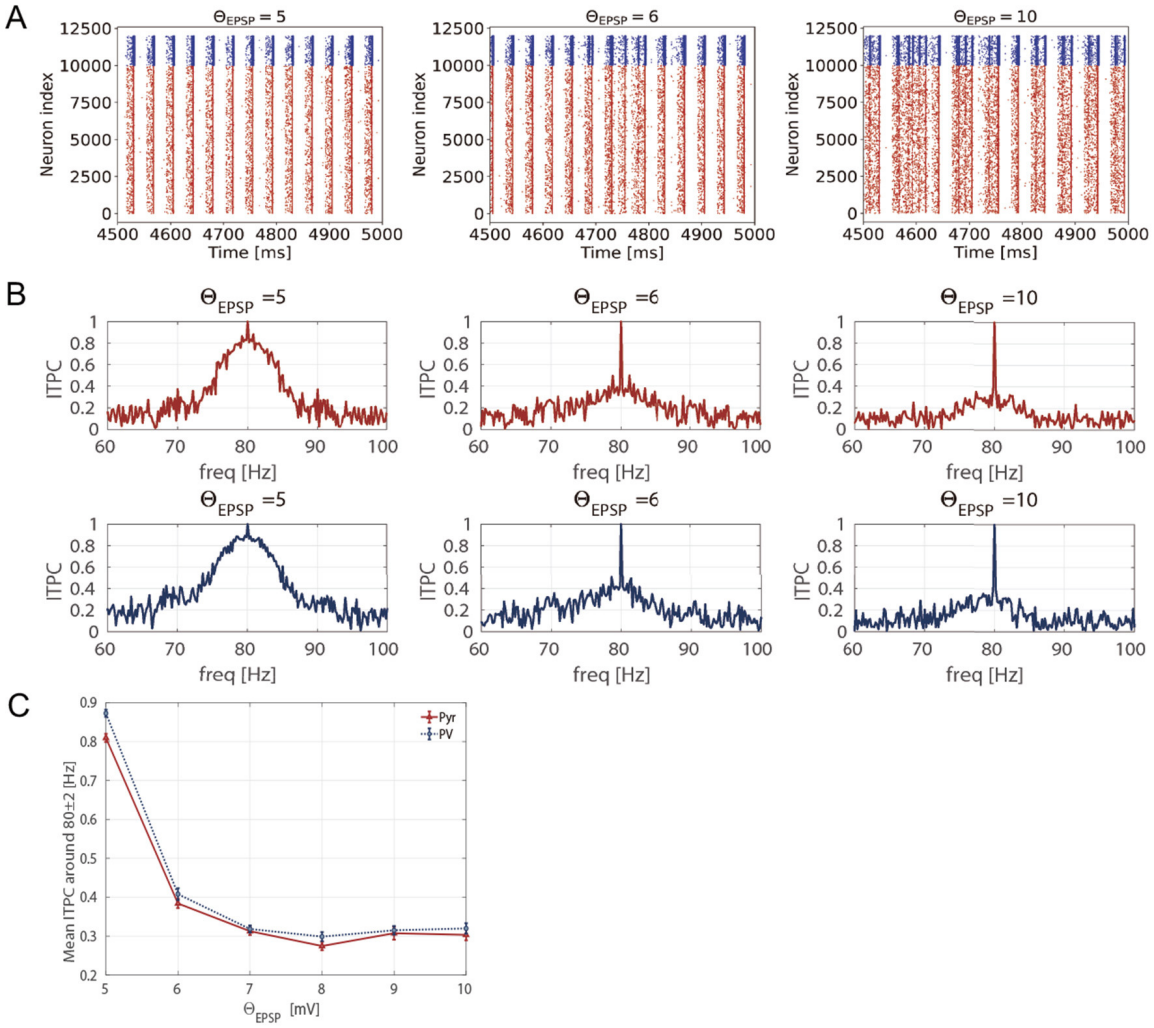
Inter-trial phase coherence (ITPC) of the firing-rate time series of excitatory pyramidal (Pyr) neuron and parvalbumin-positive (PV) interneuron populations and raster plots showing spike trains of Pyr and PV neurons in the different EPSPs thresholds (5 ≤ Θ_EPSP_ ≤ 10 [mV]). **(A)** The raster plots show spike trains of Pyr (red, index from 0 to 9,999) and PV (blue, index from 10,000 to 11,999) neurons. **(B)** The representative ITPC profiles of different EPSP thresholds Θ_EPSP_ = 5, 6, 10 [mV]. The two rows show the ITPC profile of 80 [Hz] for Pyr (red, upper) and PV (blue, lower) neuronal populations. **(C)** Mean ITPC values in the Pyr (red, triangle markers) and PV (blue, circle markers) neuron populations around the input frequency *f*_*s*_ = 80 [Hz] averaged *f*_*s*_±2 [Hz]. The markers and error bars show the mean and standard deviation across 10 evaluations.

To determine whether excessively enhanced spontaneous activity reduces neuronal activity responsiveness, we calculated the ITPC values at different EPSP thresholds. Specifically, we changed the EPSPs threshold Θ_EPSP_ from 5 to 10 [mV] within a biologically plausible range (Lefort et al., 2009). Figure 7A shows the stimulus-evoked spiking activity at 80 [Hz] for Pyr and PV neuronal populations at different EPSP thresholds (Θ_EPSP_ = 5, 6, 10 [mV]). Figure 7B shows the ITPC profiles for different EPSP threshold values. The ITPC value near the input frequency (80 ± 2 [Hz]) decreased significantly at the EPSPs threshold of Θ_EPSP_ = 6, 10 [mV]. As illustrated in Figure 7C, the mean ITPC values also significantly decreased (< 0.5) when the EPSPs threshold was Θ_EPSP_ ≥ 6 [mV], compared to the enhanced consistency of neuronal responsiveness shown in Figure 3C ($G_{k}^{\text{IE}}=0.0027$ at 80 [Hz]). This suggests that a few large EPSPs induce excessively enhanced spontaneous activity, resulting in the disruption of the neuronal response to input stimuli.

## 4 Discussion

In this study, we validated the hypothesis that the neural population becomes more responsive and exhibits consistent responses to external stimuli at gamma-band frequencies due to the maturation of the GABAergic system, which underlies the onset of CP plasticity (Hensch, 2004, 2005; Toyoizumi et al., 2013; Takesian and Hensch, 2013; Wong-Riley, 2021). This hypothesis arises from the theory that mature inhibition increasingly suppresses stimulus-irrelevant spontaneous activity, which enhances stimulus-evoked responses (Toyoizumi et al., 2013). To quantify the neural network response, we measured the ITPC values, which represent the consistency of the phase of neural activity over trials. We showed that the coherence of stimulus-evoked neuronal activity at 80 [Hz] was significantly enhanced as the inhibition matured. The profile of the mean ITPC values showed an inverted U-shape relative to the developmental increase in inhibition, with the peak of this profile showing higher coherence (mean ITPC ≥ 0.8). This result suggests the existence of an optimized E/I balance that maximizes the consistency of the neural responses in our SNN. Additionally, our results indicated that moderate spontaneous activity contributes to the enhancement of stimulus-evoked neuronal responses with increased inhibition, while also suggesting frequency selectivity.

### 4.1 The contribution of E/I balance and maturational process of inhibition to the higher coherence of neuronal response at gamma-band frequency

First, we discuss how the maturation of inhibition enhances the coherence of gamma-band neuronal population responses to stimuli and how this underlies synaptic plasticity. Our modeling study showed that the ITPC values in response to gamma-band periodic inputs were strengthened by the suppression of spontaneous activity due to the maturation of inhibition. Our findings are consistent with previous research by Toyoizumi et al. (2013) with respect to the neuronal response becoming more externally driven because of the suppression of spontaneous activity caused by the maturation of inhibition. This effect is also supported by *in vivo* experiments by Fang et al. (2021) where an enhanced signal-to-noise ratio in visual responses was observed. In addition to the transition of neuronal responses depending on inhibition levels, a relationship between enhanced externally driven neuronal responses and plasticity mechanisms has been demonstrated (Toyoizumi et al., 2013). Specifically, the maturation of inhibition facilitates Hebbian plasticity in a single pyramidal neuron model, contributing to higher responsiveness to external input. The fundamental principle of Hebbian plasticity, a major form of synaptic plasticity, is that the correlated activities of pre-synaptic and postsynaptic neurons drive the strengthening of specific synapses (Turrigiano, 2008; Toyoizumi et al., 2014). In other words, consistent neuronal activity in response to external stimuli reflects enhanced synaptic strength. In our study, the response of the neuronal population to external stimuli was investigated using ITPC analysis. This analysis revealed that the higher ITPC values (see Figure 3C), which indicated consistent neuronal activity across trials, suggest heightened plasticity at the neuronal population level.

Next, we discuss the reasons for the remarkable frequency selectivity of the inhibition levels observed during our evaluation. Our results demonstrate that the ITPC value at 80 [Hz] was significantly enhanced due to the maturation of inhibition, whereas a similar ITPC profile was not observed at 40 [Hz]. This frequency selectivity was demonstrated in neurophysiological studies using electroencephalography (EEG) (Tsuchimoto et al., 2011; Tada et al., 2021). For example, a study on abnormalities in the neural circuitry in patients with schizophrenia revealed that the ASSR-PLF (phase-locking factor), which measures phase-locked activity similar to the ITPC values of stimulus-evoked neural activity, showed significant changes at frequencies of 40 [Hz] and 80 [Hz], while similar characteristics were not observed for other frequency bands (Tsuchimoto et al., 2011). Similarly, a study that elucidated the frequency-specific characteristics of the oscillatory activity demonstrated that the ITPC at 40 [Hz] was prominent across a wide range of stimulus frequencies (20, 30, 40, 60, 80, 120, *and*160 [Hz]) (Tada et al., 2021). Moreover, the ITPC at specific frequencies in patients with psychiatric disorders was lower than that in healthy controls, reflecting abnormal evoked oscillatory activity (Grent et al., 2023; Wolff and Northoff, 2024). This disruption of consistent responses to external stimuli suggests an E/I imbalance due to the excessive excitability of pyramidal neurons and weakened cortical inhibition (Tatti et al., 2017; Grent et al., 2023). In addition, the frequency selectivity of neural activity depends on the resonance frequency of the neural circuit (Galambos et al., 1981; Pastor et al., 2002). Therefore, our results show that a difference in neuronal responses to 40 [Hz] and 80 [Hz] inputs (see Figure 3) is consistent with the experimental findings. However, a more detailed theoretical analysis, such as examining the time constants at the population level in the SNN, is necessary to elucidate the frequency selectivity in our model (Teramae and Fukai, 2007).

Furthermore, we discuss the disruption of neuronal response coherence caused by E/I imbalance due to other excitable factors in neural circuits instead of manipulating inhibition. As previously discussed, our results showed that stimulus-evoked neuronal coherence was enhanced at specific inhibition levels. This indicates the presence of an optimal E/I balance necessary for achieving stabilized neuronal coherence in response to external stimuli. Moreover, we evaluated the influence of the E/I balance on neuronal responses from a perspective distinct from the increase in inhibition. Specifically, we increased the excitation levels of the neural circuit by changing the threshold of EPSPs from 5 [mV] to 10 [mV]. Consequently, excessive excitatory neural activity due to the increase of large EPSPs reduced neuronal coherence (see Figure 7). This may be consistent with previous findings regarding the E/I imbalance induced by hyperexcitability of neuronal circuits (Obi-Nagata et al., 2023). It has been reported that overrepresentation of strong EPSPs can lead to increased neuronal firing, resulting in working memory impairment or hallucinations in patients with schizophrenia (Obi-Nagata et al., 2023). This suggests that the stability of neuronal activity is disrupted by an E/I imbalance due to excessive excitatory neural activity. Taken together, the neuronal population responses are influenced by the E/I balance, which is determined not only by the level of inhibition of $G_{k}^{\text{IE}}$ but also by the excitation levels due to large EPSPs.

### 4.2 Modeling post-CP in neural networks: manipulating glutamatergic neurotransmission onto PV interneruons

The post-CP conditions of the neural network model were considered. When the CP closes, PV interneurons become especially encased in an extracellular matrix (ECM) known as perineuronal nets (PNNs) (Fawcett et al., 2019; Reichelt et al., 2019; Carceller et al., 2023; Hug and Mpai, 2024). PNNs limit further increases in neuronal plasticity and trigger the closure of the CP. Although accumulating evidence has demonstrated that PNNs are hallmarks of the post-CP, their effects on synaptic and circuit mechanisms remain unclear. It has been suggested that the accumulation of PNNs around PV cells is associated with reduced glutamatergic neurotransmissions in PV interneurons (Faini et al., 2018). In other words, further changes in the E/I ratio of PV cells trigger the closure of CP (Faini et al., 2018). Based on these findings, we investigated the effect of reduced synaptic weights from Pyr to PV neurons on neuronal coherence. Specifically, we changed the E/I balance from the maximum neuronal response setting. Our results indicate that the reduction in glutamatergic neurotransmissions to PV interneurons disrupts neuronal coherence due to an E/I imbalance and leads to neural excitation (see Figure 5). In this study, we assumed that a more consistent response to external inputs would indicate increased neural plasticity; therefore, these results suggested diminished synaptic plasticity. Furthermore, Faini et al. (2018) also suggested that PNN accumulation during post-CP development reduced the strength of glutamatergic thalamic input to PV interneurons. Our results demonstrated that the reduction of the input strength causes a decrease in the mean ITPC (see Figure 6). Namely, we found that the accumulation of PNNs regulates neuronal responsiveness through the modulation of the E/I ratio within the network and the control of the strength of external stimuli in the SNN model. The mechanisms and functions of PNN have been investigated from various perspectives at the molecular and neural circuit levels (Giamanco and Matthews, 2012; Devienne et al., 2021). Although Our model does not capture the detailed and plausible biological findings of PNNs, we assume that our proposed approach, which focuses on glutamatergic neurotransmissions to PV interneurons, can be utilized to model the post-CP condition in the SNN.

### 4.3 Limitations and future directions of this study

Finally, we explore the limitations of the proposed model. In the present study, we calculated the ITPC values in the SNN model to investigate the consistency of neuronal responses to stimulus-evoked activity with respect to the maturation of the inhibition underlying CP plasticity. Our results suggest that the moderate maturation of inhibition contributes to neural activity being more responsive and consistent with external inputs, suggesting heightened synaptic plasticity during CP. However, it is crucial to elucidate how our findings-specifically, that neuronal activity responses become more consistent with external inputs in the SNN-actually contribute to plasticity mechanisms. To confirm the relationship between synaptic plasticity and neuronal phase coherence in a computational model, we should incorporate synaptic plasticity, such as Hebbian and homeostatic plasticity mechanisms, into our current model in future studies (Turrigiano, 2008; Toyoizumi et al., 2014). Notably, in our study, the maturation of inhibition influenced the ITPC values but did not affect the power. However, an increase in the gamma power induced by stimuli during CP may be a signature of plasticity (Quast et al., 2023). This discrepancy in previous research may be attributed to the fact that our SNN model did not involve plasticity. In addition, we used only PV interneurons as GABAergic neurons in the present study. However, to better understand the roles of inhibitory neurons, future models should incorporate other types of inhibitory neurons, such as somatostatin-positive (SST) cells and vasoactive intestinal polypeptide-positive (VIP) interneurons (van Versendaal and Levelt, 2016; Lee and Mihalas, 2017). Additionally, incorporating other types of neurons and interconnections among brain regions and cortical columns generates other frequency band oscillations (Kopell et al., 2000; Cohen, 2014). Such biologically plausible modeling is also valuable for analyzing neuronal activity at other frequencies (Başar et al., 2001; Colgin, 2013). Furthermore, as discussed previously, we could not clarify the dynamic mechanism of frequency selectivity at different inhibition levels. This issue must be revealed through theoretical analysis, such as mean-field analysis (Teramae and Fukai, 2007; Yu and Taillefumier, 2022). Finally, in addition to focusing on glutamatergic neurotransmissions in PV interneurons, we should develop a more plausible post-CP model to understand the precise functions of PNNs.

## 5 Conclusion

To reveal how the maturational process of the GABAergic system contributes to a consistent response of stimulus-evoked neural activity at gamma-band frequencies, we calculated the ITPC values for different synaptic weights of neurons at the neuronal population level using the SNN model with long-tailed EPSPs. The neuronal response coherence to the external periodic input exhibited an inverted U-shape with respect to the maturation of inhibition. Furthermore, the peak of this profile was consistent with the moderate suppression of the gamma-band spontaneous activity. Thus, our findings indicated that the optimized E/I balance associated with heightened synaptic plasticity is related to a consistent response to stimulus-evoked neural activity. Consequently, this regulated neural network contributes to neuronal activity and is externally driven to adapt to the environment. Our study has several limitations, but computational modeling can contribute to understanding the underlying mechanisms that maximize synaptic plasticity at the neuronal population level by investigating neuronal coherence in response to external stimuli.

## Data Availability

The original contributions presented in the study are included in the article/Supplementary material, further inquiries can be directed to the corresponding author.
