# Supplementary material for: Neural activity responsiveness by maturation of inhibition underlying critical period plasticity
Source: Front Neural Circuits. 2025 Jan 22;18:1519704. doi: 10.3389/fncir.2024.1519704 (PMC11794837; doi:10.3389/fncir.2024.1519704)
Supplement: Supplementary file 1 [file Data_Sheet_1.pdf]

Supplementary Material: Neural Activity Responsiveness by Maturation of Inhibition Underlying Critical Period Plasticity

1 DEFINITION OF PERIODIC SPIKE TRAINS

In our spiking neural network (SNN), a periodic spike train was applied to investigate the stimulus-evoked response of the neuronal activity induced by periodic inputs, known as the steady-state response (SSR) (Regan, 1966). This spike train was generated by spiking neurons ($\mathrm{N_{in}} = 100$) using the integrate-and-fire (IF) model. The membrane potential of the input neurons, $v_{\mathrm{in}}(t)(i = 1, 2, ..., 100)$ is defined as follows:

$$\frac{dv_{\mathrm{in}}}{dt} = \frac{1}{\tau_{\mathrm{in}}}(-v_{\mathrm{in}} + \sin(2\pi f_s t) + b), \tag{S1}$$

$$\text{if } v_{\mathrm{in}}(t) \geq 1, \text{ then } v_{\mathrm{in}}(t) = 0, \tag{S2}$$

where τ_{in} and b denote the decay constant of the membrane ($\tau_{\mathrm{in}} = 20$ [ms]) and the constant current, respectively. As shown in Eq. (S1), the neurons fire following a sinusoidal current $\sin(2\pi f_s t)$. In this study, we focused on gamma-band neural activity; therefore, we employed $f_s = 40$ [Hz] and 80 [Hz] as periodic inputs. When evaluating these frequency inputs, parameter b was adjusted to achieve the same firing rate; this was, specifically, a total firing rate of approximately $5,000$ [Hz] in the neural population. The values of b for the input frequencies of 40 [Hz] and 80 [Hz] are set to 1.32 and 0.95, respectively. Figure S1 shows raster plots of the spike trains of the input neurons. For the generation of input stimuli, we referred to examples on Brian2 (https://brian2.readthedocs.io/en/stable/examples/phase_locking.html) (Goodman et al., 2014). The spikes at time s_i generated by this equation are inputted into the SNN followed by $\sum_i W^{\mathrm{in}}_{j,i} \sum_{s_i} \delta(t - s_i)$ in Eq. (1). Here, $W^{\mathrm{in}}_{j,i}$ and s_i are the synaptic weights of the jth neuron in the SNN from ith input neuron and the spiking time of the input from the ith neuron, respectively. In this study, the connectivity between the input and the neurons of the SNN was random, and the coupling probability was 0.25. Additionally, $\delta(t)$ is a delta function; therefore, when the ith input neuron is fired at time $t = s_i$, the membrane potentials of the neurons of the SNN are increase with the voltage $W^{\mathrm{in}}_{j,i}$ [mV]. In this study, we set $W^{\mathrm{in}}_{j,i} = 0.5$ [mV].

2 SIMULATION SETTING FOR INTER-TRIAL PHASE COHERENCE ANALYSIS

In the present study, we evaluated neuronal responses over 5 [s] and calculated the Inter-Trial Phase Coherence (ITPC) value across 100 trials. To investigate the validity of these simulation settings, we compared them with different settings at a periodic input frequency of 80 [Hz].

2.1 Evaluation Period

First, we compared the evaluation periods of 5, 10, and 15 [s]. Regardless of the evaluation period, the same characteristic, an inverted U-shape indicating a response to external input, was observed, as shown in Fig. S2. In addition, the ITPC value at approximately 80 ± 2 [Hz] for the 5 [s] was higher than that of the others. Therefore, we use 5 [s] as the evaluation period.

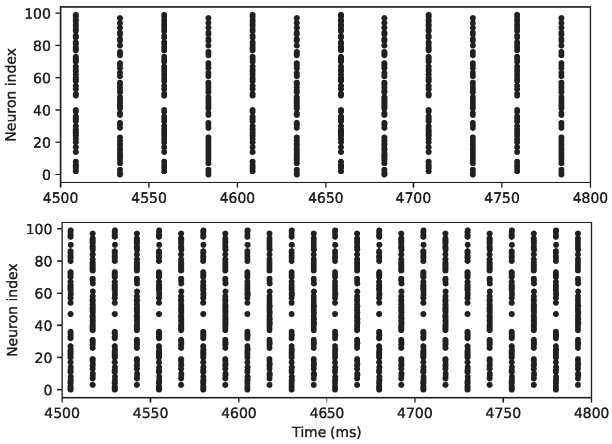

Figure S1. Raster plots showing spike trains of input neurons behaving periodically at 40 [Hz] (upper) and 80 (lower) [Hz].

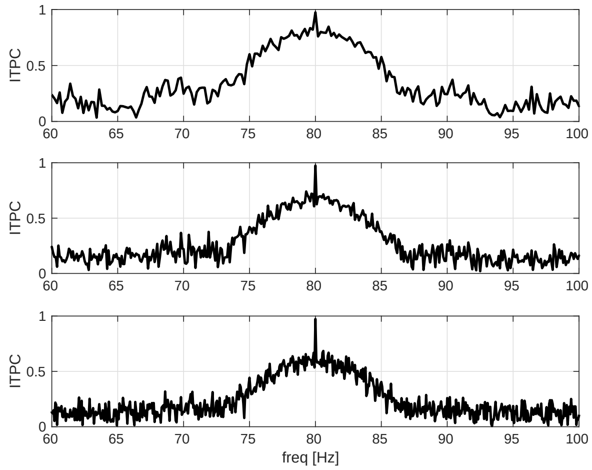

Figure S2. Inter-trial phase coherence (ITPC) of the firing-rate time series of excitatory pyramidal neuron populations (r_{E}) in the case of input frequency 80 [Hz] and $G_k^{\mathrm{IE}} = 0.0027$. Three rows show the evaluation durations of 5 (top), 10 (middle), and 15 (bottom) [s], respectively.

2.2 The Number of Trials for Calculating Inter-Trial Phase Coherence Values

Second, we compared the ITPC values among the cases with number of trials $T = 10, 30, 50, 100, 150, 200, 250$ trials in Eq. (6). Importantly, a random resampling was performed for each trial from 300 trials. Sampling of the population data was repeated 10 times, and the mean ITPC value and standard deviation were calculated. During this evaluation, the mean ITPC converged in $T = 100$ trials, as shown in Fig. S3. Consequently, we decided to use 100 trials to calculate the ITPC values. The mean ITPC converges at $T = 100$ trials, but the standard deviation decreases. In the following paragraph, we discuss why the standard deviation of ITPC decreases as the number of trials increases.

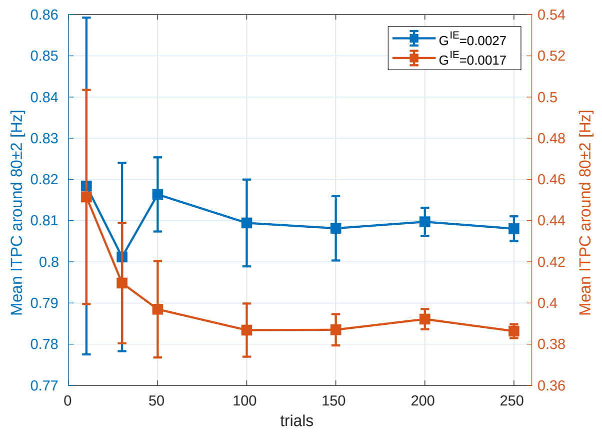

Figure S3. Inter-trial phase coherences (ITPC) values across different trial numbers ($T = 10, 30, 50, 100, 150, 200, 250$ trials in Eq. (6) in different inhibition levels $G_k^{\mathrm{IE}} = 0.0017, 0.0027$. The mean ITPC value converged at $T = 100$ trials at both inhibition levels. Since ITPC is not an unbiased estimator, the standard deviation decreases with increasing T.

We now describe the dependence of the number of trials on the standard deviation of ITPC. First, ITPC is defined as

$$\mathrm{ITPC}(T, f) = \left| \frac{1}{T} \sum_{j=1}^{T} \frac{F_j(f)}{|F_j(f)|} \right| \tag{S3}$$

$$= \left| \frac{1}{T} \sum_{j=1}^{T} e^{i\phi_j(f)} \right|, \tag{S4}$$

where T is the number of trials and f is the Fourier frequency. $F_j(f)$ is the Fourier component at frequency f in the jth trial. $\phi_j(f)$ denotes the phase of the component. As ITPC is not an unbiased estimator, it is necessary to use a sufficiently large T to reduce bias and variance. In the following section, we examine how the number of trials T affects the bias and standard deviation of ITPC.

When ITPC is applied to random noise, the phase ϕ_j is expected to be random. Therefore, we model ϕ_j as an independent random variable that is uniformly distributed over the interval $[0, 2\pi]$. Let z denote the ITPC in this scenario. We can express z as

$$z = \sqrt{A^2 + B^2}, \tag{S5}$$

$$A = \frac{1}{T} \sum_{j=1}^{T} \cos(\phi_j), \tag{S6}$$

$$B = \frac{1}{T} \sum_{j=1}^{T} \sin(\phi_j). \tag{S7}$$

Variables A and B have the following properties.

$$\mathbb{E}[A] = 0,\ \mathbb{E}[B] = 0, \tag{S8}$$

$$\sigma_A = \frac{1}{\sqrt{2T}},\ \sigma_B = \frac{1}{\sqrt{2T}}, \tag{S9}$$

where $\mathbb{E}[\cdot]$ denotes the expected value and σ_X is the standard deviation of X, which is defined as $\sigma_X = \sqrt{\mathbb{E}[(X - \mathbb{E}[X])^2]}$. For a large T, the central limit theorem implies that A and B are approximately Gaussian distributions.

$$A \sim N(0, \frac{1}{2T}),\ B \sim N(0, \frac{1}{2T}), \tag{S10}$$

where $N(a, b)$ denotes a Gaussian distribution with mean a and variance b. To demonstrate that A and B are not correlated. We calculate their covariance:

$$\mathbb{E}[AB] = \frac{1}{T^2}\mathbb{E}\left[\sum_{i=1}^{T}\sum_{j=1}^{T}\cos(\phi_i)\sin(\phi_j)\right] \tag{S11}$$

$$= \frac{1}{T^2}\sum_{i=1}^{T}\sum_{j=1}^{T}\mathbb{E}\left[\cos(\phi_i)\sin(\phi_j)\right] \tag{S12}$$

$$= 0. \tag{S13}$$

Moreover, because A and B are multivariate Gaussian distributions (for large T), a lack of correlation implies independence.

Therefore, the random variable z follows a Rayleigh distribution with the scale parameter $\beta = \sqrt{\frac{1}{2T}}$. Finally, the expected value and standard deviation of z are given by:

$$\mathbb{E}[z] = \beta\sqrt{\frac{\pi}{2}} = \frac{1}{2}\sqrt{\frac{\pi}{T}},\ \sigma_z = \beta\sqrt{\frac{4-\pi}{2}} = \frac{1}{2}\sqrt{\frac{4-\pi}{T}}. \tag{S14}$$

This result demonstrates how the bias ($\mathbb{E}[z]$) and standard deviation (σ_z) of ITPC decrease with an increasing number of trials T when the underlying phases are random.

3 POWER SPECTRUM ANALYSIS FOR STIMULUS-EVOKED NEURAL ACTIVITY

In this study, we calculated the power spectra of the spontaneous activity from the firing-rate time series of the excitatory pyramidal neural population $r_{\mathrm{E}}(t)$, as detailed in the main text. Similarly, we calculated the power spectra of the stimulus-driven activity at 40 [Hz] and 80 [Hz]. Figure S4 presents the power spectral profiles at these frequencies. We did not observe an increase in power at either frequency as inhibition matured. In particular, the ITPC value at 80 [Hz] was enhanced by the inhibition level (see Fig. 3B); however, the power at this frequency was not affected. However, an increase in the gamma power induced by stimuli during CP may be a signature of plasticity (Quast et al., 2023). In mice, a brief peak in gamma power during CP emerged in V1. This transient gamma oscillation reflects plastic changes in thalamocortical-to-PV synaptic strength, which presents a discrepancy between our computational model

and physiological experiments. This discrepancy may be due to the fact that the SNN model does not consider plasticity.

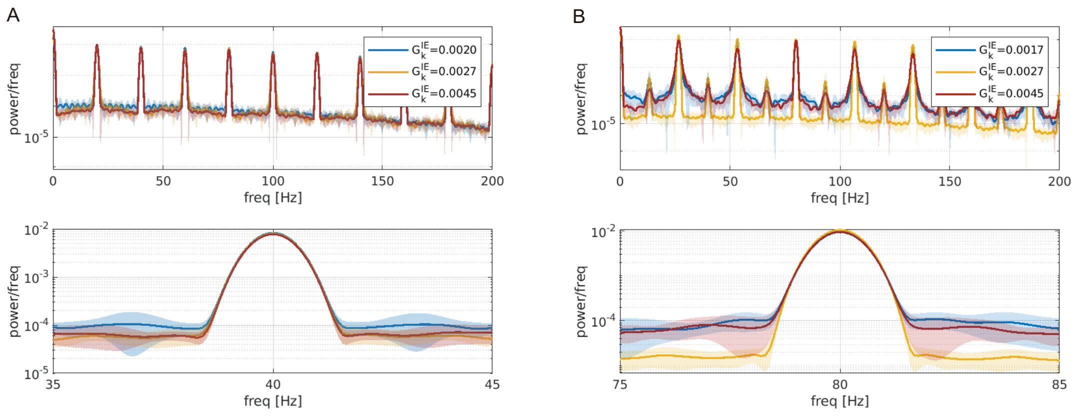

Figure S4. Power spectra of the firing-rate time series of the excitatory pyramidal neural population ($r_{\mathrm{E}}(t)$) driven by periodic inputs of 40 [Hz] and 80 [Hz] with the threshold for EPSPs distribution set at 5 [mV] ($\Theta_{\mathrm{EPSP}} = 5$). Each color corresponds to the results of ITPC as shown in Fig. 3B. (A) The profile of 40 [Hz] in the range $[0, 200]$ [Hz] (upper) and the magnified in the range around $f_s \pm 5$ [Hz] (lower). (B) The profile of 80 [Hz] in the range $[0, 200]$ [Hz] (upper) and the magnified in the range around $f_s \pm 5$ [Hz] (lower). The solid and shaded areas show the mean and standard deviation over 10 trials.

REFERENCES

Goodman, D. F., Stimberg, M., Yger, P., and Brette, R. (2014). Brian 2: neural simulations on a variety of computational hardware. *BMC neuroscience* 15, 1–1

Quast, K. B., Reh, R. K., Caiati, M. D., Kopell, N., McCarthy, M. M., and Hensch, T. K. (2023). Rapid synaptic and gamma rhythm signature of mouse critical period plasticity. *Proceedings of the National Academy of Sciences* 120, e2123182120

Regan, D. (1966). Some characteristics of average steady-state and transient responses evoked by modulated light. *Electroencephalography and clinical neurophysiology* 20, 238–248
